# CD137 (4-1BB) Signalosome: Complexity Is a Matter of TRAFs

**DOI:** 10.3389/fimmu.2018.02618

**Published:** 2018-11-15

**Authors:** Juan M. Zapata, Gema Perez-Chacon, Pablo Carr-Baena, Ivan Martinez-Forero, Arantza Azpilikueta, Itziar Otano, Ignacio Melero

**Affiliations:** ^1^Instituto de Investigaciones Biomédicas “Alberto Sols” (CSIC-UAM), Madrid, Spain; ^2^Instituto de Investigación Hospital Universitario La Paz, Madrid, Spain; ^3^Departamento de Inmunologia and Inmunoterapia, Centro de Investigación Medica Aplicada, Universidad de Navarra, Pamplona, Spain; ^4^MSD, London, United Kingdom; ^5^Departamento de Inmunologia e Inmunoterapia, Clinica Universitaria, Universidad de Navarra, Pamplona, Spain; ^6^Instituto de Investigacion Sanitaria de Navarra, Pamplona, Spain; ^7^Centro de Investigación Biomédica en Red Cáncer (CIBERONC), Madrid, Spain

**Keywords:** CD137, 4-1BB, TNFR, TRAF1, TRAF2, TRAF3, Immunotherapy, cytotoxic T lymphocytes (CTL)

## Abstract

CD137 (4-1BB, Tnsfr9) is a member of the TNF-receptor (TNFR) superfamily without known intrinsic enzymatic activity in its cytoplasmic domain. Hence, akin to other members of the TNFR family, it relies on the TNFR-Associated-Factor (TRAF) family of adaptor proteins to build the CD137 signalosome for transducing signals into the cell. Thus, upon CD137 activation by binding of CD137L trimers or by crosslinking with agonist monoclonal antibodies, TRAF1, TRAF2, and TRAF3 are readily recruited to the cytoplasmic domain of CD137, likely as homo- and/or heterotrimers with different configurations, initiating the construction of the CD137 signalosome. The formation of TRAF2-RING dimers between TRAF2 molecules from contiguous trimers would help to establish a multimeric structure of TRAF-trimers that is probably essential for CD137 signaling. In addition, available studies have identified a large number of proteins that are recruited to CD137:TRAF complexes including ubiquitin ligases and proteases, kinases, and modulatory proteins. Working in a coordinated fashion, these CD137-signalosomes will ultimately promote CD137-mediated T cell proliferation and survival and will endow T cells with stronger effector functions. Current evidence allows to envision the molecular events that might take place in the early stages of CD137-signalosome formation, underscoring the key roles of TRAFs and of K63 and K48-ubiquitination of target proteins in the signaling process. Understanding the composition and fine regulation of CD137-signalosomes assembly and disassembly will be key to improve the therapeutic activities of chimeric antigen receptors (CARs) encompassing the CD137 cytoplasmic domain and a new generation of CD137 agonists for the treatment of cancer.

## Brief introduction to the TRAF protein family

TNF Receptor Associated Factors (TRAFs) are a family of 6 proteins (TRAF1 to 6) characterized for having a protein region composed by a coiled coil followed by a seven-eight anti-parallel β-sheets at the C-terminus of the protein forming what has been coined as the TRAF domain (TD) (1, 2). This domain is also known as the Meprin and TRAF-C homology domains (MATH), since meprins, a family of extracellular proteases, also have a protein domain with high homology to the TD (3). In addition, there are also 3 proteins in humans encompassing internal *bona fide* TRAF domains: tripartite motif (TRIM)-37, ubiquitin specific protease (USP)-7 and speckle-type POZ protein (SPOP) (4). Of note is that there is a protein known as TRAF7 that lacks a TD but has a RING and zinc finger domains similar to those of some members of the TRAF family proteins (5) and whose membership to the TRAF family is controversial.

TRAF1 to 6 were first identified as TNF-Receptor (TNFR) binding proteins, but it soon become evident that different members of the TRAF family were also involved in the regulation of pattern recognition receptors, including members of the Toll-like receptors (TLRs), nucleotide-binding oligomerization domain (NOD)-like receptors (NLRs) and retinoic acid-inducible gene (RIG)-1-like Receptors (RLRs), thus demonstrating the key role of TRAF family proteins in the regulation of both innate and adaptive immunity [Reviewed by (6)]. Moreover, some members of the TRAF family also regulate cytokine receptors (6, 7). A role for TRAF family members in development has also been described (8–10).

TRAFs are the molecules that first engage the activated TNFR and act as scaffold proteins recruiting other proteins, including kinases, ubiquitin ligases and deubiquitinases among other regulatory proteins to conform the TNFR-signalosome. TRAF family members, with the exception of TRAF1, have a RING finger domain that endows some of them with the capacity to act as E3 ubiquitin ligases. Thus, TRAFs can ubiquitinate different components of the signalosome, including the TRAFs themselves, and modulate the activity of the complex (6).

**Figure 1:**
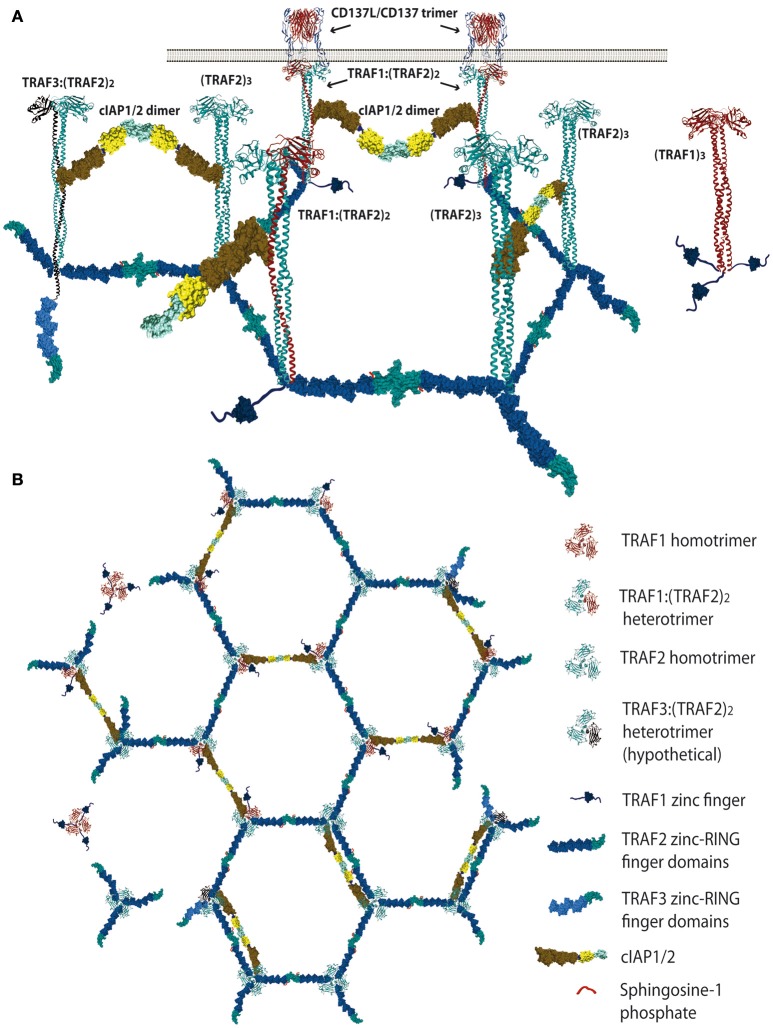
Schematic representation of the proposed TRAF trimer configurations and interactions in the CD137L/CD137 hexagonal lattice. **(A)** Lateral view representing the various TRAF-trimer configurations that could be recruited to the activated CD137 trimers. The figure also shows the TRAF2-RING finger dimers that would likely be formed between the RING finger domains of two TRAF2 molecules from adjacent trimers, which is a requirement for E3 ubiquitin ligase activity. Similar interactions between the RING domains of cIAP1/2 from contiguous trimers are also expected. **(B)** It is show in top view how the CD137-recruited TRAF trimers would arrange forming a large hexagonal network that would be stabilized by the establishment of RING finger domains dimers between the TRAF2 molecules from adjacent trimers or between the RING finger domains of contiguous cIAP1/2 molecules. Further explanation in the text. Protein structure coordinates were obtained from the PDB database and molecular graphics were performed with UCSF Chimera (15).

There is a redundancy in the ability of different members of the TRAF family to interact with similar TRAF-binding peptidyl regions located in the cytosolic tails of the TNFRs [reviewed in (1, 2, 11)]. Moreover, besides this critical binding region, the surrounding amino acids to the peptide core motif in the cytosolic tail of TNFRs might also provide structural constrains that may have an effect on the binding affinity. In addition, the crystal structures of TDs bound to the cytosolic region of distinct TNFR family members have shown that particular structural features of the TD of each TRAF family member, in particular of those forming the TNFR-binding crevice, are critical in determining their specificities and binding affinities to the TNFRs [reviewed in (12)]. Altogether, these differences determine the binding specificity and affinity of the members of the TRAF family for the different TNFR family members (1, 11–14). Therefore, it is expected that a competition would be established between different TRAFs trimers to dock at the ligand-activated TNFR trimer, raising the possibility that neighboring TNFR trimers in the very same cell will hold TRAF trimers with different configurations. In addition, some TRAF family members can form heterotrimers (see below and Figure 1), adding further complexity to the system. Consequently, the composition of the signalosome mounted by each member of the TNFR family is likely to be highly influenced by the recruited TRAF family members. Besides, the signalosome composition would likely be cell type and activation state dependent, as it will be contingent on the expression levels and subcellular localization of the different proteins that could be part of this complex.

## CD137 as a model of how TRAFs configure a TNFR-signalosome

CD137 (4-1BB, TNFSFR9) is one of the TNFRs having a more restricted number of TRAF family members involved in its regulation, since only TRAF1, TRAF2, and TRAF3 interact with and control CD137 activity. CD137 is a member of the TNFR family whose expression is highly induced in CD8 T and NK lymphocytes upon activation, where it works as a critical costimulatory receptor (16–18). Moderate to low levels of CD137 expression could also be found in other activated immune populations, including CD4 T cells, B cells, monocytes, macrophages, granulocytes and dendritic cells and, in these cells, CD137 can also convey costimulatory signals (17, 19).

CD137 delivers potent costimulatory signals to the activated CTLs and memory T cells promoting cell proliferation and survival and also endowing CD8 T cells with CTL effector functions. As such, in the last 15 years, CD137 has become one of the most exciting targets to enhance anti-cancer immunity for its ability of boosting CTLs with anti-tumor effector functions (20–22).

CD137 binds to CD137-Ligand (CD137L, 4-1BBL, or tnfsf9), a member of the TNF superfamily (TNFSF). CD137L is mostly expressed on macrophages, activated B cells, and dendritic cells (23). In this regard, it is noteworthy that antigen presenting dendritic cells in tumors and tumor draining lymph nodes and tumor associated macrophages seem to be responsible for providing CD137L to cytotoxic T lymphocytes (CTLs) migrating to tumors (24). CD137L remains the sole intercellular ligand known for CD137, but binding of CD137 to extracellular matrix proteins, such as fibronectin, vitronectin, laminin and collagen VI (25) has been reported, albeit functional consequences of the binding to these additional putative ligands remain unknown. Interestingly, binding of CD137 to galectin-9, a member of the β-galactoside–binding family of lectins, has also been shown (26). Interestingly, galectin-9 binding to CD137 does not interfere with the binding of either CD137L or agonistic anti-CD137 mAbs to the receptor. Instead, it positively regulates CD137 function by keeping preassembled CD137 complexes together (26), which could be then further cross-linked by CD137L or by anti-CD137 mAbs.

The crystal structure of the CD137L trimer shows distinctive structural features that differ from those of other TNF family members. In this regard, CD137L trimer resembles a three-bladed propeller which is different from the cork-like shape of the trimers of other members of the family (27). This shape also confers some structural particularities to CD137/CD137L complex, which folds as a windmill-like shape structure. Despite these structural differences with other TNF and TNFR family members, these results are still fully consistent with a model for CD137/CD137L interaction similar to that of other members of the TNFR family, in which a trimeric ligand binding to three receptors conforms the basic unit of signaling (28, 29).

As for many other members of the TNFRSF, CD137 uses TRAFs as scaffold proteins to build its signalosome. CD137 has been found to bind to TRAF1, TRAF2, and TRAF3 (30–32) through two poly-acidic TRAF-binding consensus regions located in its cytosolic tail _234_TTQEE_238_ and _246_PEEEE_250_, which are similar to those found in other TNFR family members [reviewed in (1, 2)]. Point mutations studies showed that all three TRAFs seem to have binding preferences for the C-terminal _246_PEEEE_250_ TRAF-binding region, suggesting that they might compete with each other for interacting with the activated receptor (31). Due to the proximity of the two TRAF binding sites, binding of one TRAF trimer to one of these regions, would render the other region unavailable by steric hindrance. However, this does not rule out the presence of different TRAFs associated to the same activated CD137 trimer, since TRAF1 and TRAF2 form heterotrimers that can associate to the activated TNFR (33).

Cross-linking of CD137 by either CD137L (30, 34) or bivalent agonistic antibodies (35) readily results in the recruitment of TRAF1 and TRAF2 to the receptor. The involvement of both TRAF family members in the regulation of CD137 signaling and function is further confirmed by several reports showing that CD137 activity is significantly affected in model systems lacking of either TRAF1 or TRAF2 (32, 36–38). However, the role of TRAF3 as a scaffold protein building the CD137 signalosome has not been confirmed and awaits further research, although the evidence indicating the induction of NF-kB2 activation by CD137 (38) implies that TRAF3 should be directly or indirectly recruited to the CD137 signalosome (see below). In addition, recent evidence shows that TRAF3, as well as TRAF1 and TRAF2, are essential for the activity of CD137-based chimeric antigen receptors (CARs) (39), further supporting TRAF3 role in CD137 function.

The absence of a RING finger domain in TRAF1 indicates that it lacks any E3 ubiquitin ligase activity and no other intrinsic enzymatic activity for TRAF1 has been identified so far (6, 40). However, TRAF1 interacts with and regulates the activity of a variety of ubiquitin ligases and proteases (33, 41, 42) and it plays critical roles in the regulation of several members of the TNFR family [Reviewed in (43)]. Initially, since TRAF1 expression is induced upon cell activation and it has similar TNFR-binding preferences than TRAF2, it was thought that TRAF1 would work toning down TNFR signaling in activated cells by outcompeting TRAF2 from binding to the TNFRs (43). Indeed, T cells from *Traf1*-deficient mice were hyper-responsive to TNF, supporting a role for TRAF1 as a negative TNFR2 regulator (44). However, it was soon recognized that TRAF1 was not just a TRAF2 competitor but, in some instances, rather the contrary. In this regard, TRAF1 positively modulates CD40 activity by cooperating with its activity and preventing TRAF2 degradation (45, 46). In addition, TRAF1 has been also implicated in CD137-mediated survival of activated CTL (47, 48) and of memory T cells (49).

The other TRAF family members that is critical for CD137 function is TRAF2. The RING domain that TRAF2 encompasses at its N-terminus endows it with an E3 ubiquitin ligase activity. Ubiquitin-conjugating protein (Ubc)-13 (Ube2N) is thought to be TRAF2 major E2 enzyme companion, providing TRAF2 with the capacity of mediating K63-ubiquitination and subsequent activation of itself and other target proteins (50–52). In addition, TRAF2 can also catalyze K48-ubiquitination of target proteins (53, 54). Interestingly, the crystal structure of the TRAF2 RING and the first zinc finger domains described by Wu et al. (55) revealed structural constrains that would preclude Ubc13 and other related E2 ubiquitin ligases from binding to the TRAF2 RING, raising questions on the actual ability of TRAF2 to act as an E3 ubiquitin ligase. However, these discrepancies were solved when sphingosine-1 phosphate (S1P) was identified as a cofactor required for TRAF2 E3 ubiquitin ligase activity (50). Indeed, S1P seems to act as a bridge between the RING finger domain of TRAF2 and the E2 proteins. Thus, in the presence of S1P, TRAF2 was able to ubiquitinate RIP1 and itself (and/or other TRAF2 molecules in the trimer) at K63 in the presence of Ubc13 or Ubc5 (Ube2D) (50).

## CD137 signaling: a relation of known and suspected events

While TRAF2 is expressed in resting and activated T lymphocytes, TRAF1 expression is induced upon activation (60, 61). Thus, as CD137 expression will also be induced in activated T cells (16, 17), both CD137 and TRAF1 will likely coexist in activated T cells where CD137 costimulatory activity is needed for CTL expansion and for boosting effector functions. Therefore, the composition of the CD137-TRAF signaling complexes would depends on the activation state of the cell and the relative expression levels of TRAF1 and TRAF2.

The kinetics of CD137 expression in activated CD8 T cells implies that at early activation stages low levels of CD137 will be found on the T cell surface (62, 63). However, even these low levels might be sufficient to trigger CD137 signaling upon interaction with the CD137L. In this regard, it has been proposed that the ligand-free form of TNFR family members exists on the cell surface as anti-parallel dimers arranged in a two-dimensional hexagonal lattice that brings three receptor monomers together at each lattice point [Reviewed in (28)]. This model would imply that even low level of ligand-free TNFRs might be already prearranged on the cell surface in high-density spots. In the case of CD137, galectin-9 might contribute to the maintenance of these bi-dimensional hexagonal structures (26). Assuming this model, when CD137L or other TNF family member and their corresponding TNFRs come together, the ligand trimer will shift the equilibrium from the CD137 dimeric interaction to the CD137 trimeric structure. The CD137/CD137L trimers will still occupy each lattice point preserving the hexagonal structure and maintaining neighboring activated CD137 trimers close, thus facilitating the establishment of molecular interactions between adjacent trimers. TRAF trimers will be readily recruited to the activated CD137 receptor binding to the poly-acidic TRAF-binding consensus regions located in CD137 cytosolic tail. As stated above, the composition of the TRAF trimers that would be recruited to the activated CD137 will likely depend on the expression levels of the TRAF family proteins that interact with CD137, which are TRAF1, TRAF2, and TRAF3. Since TRAF1 and TRAF2 have been shown to be critical for CD137 activity it is likely that these two TRAF family members will have a major role in building the CD137 signalosome. Wu and coworkers (33) have shown that TRAF1 and TRAF2 can associate in heterotrimers, but preferentially forming a trimer with a TRAF1:(TRAF2)_2_ configuration. Therefore, a mix of TRAF1 and TRAF2 homotrimers and TRAF1:(TRAF2)_2_ heterotrimers would be recruited to the activated CD137 in a way that would depends on their amounts and specific affinities to the TNFR.

**Figure 2:**
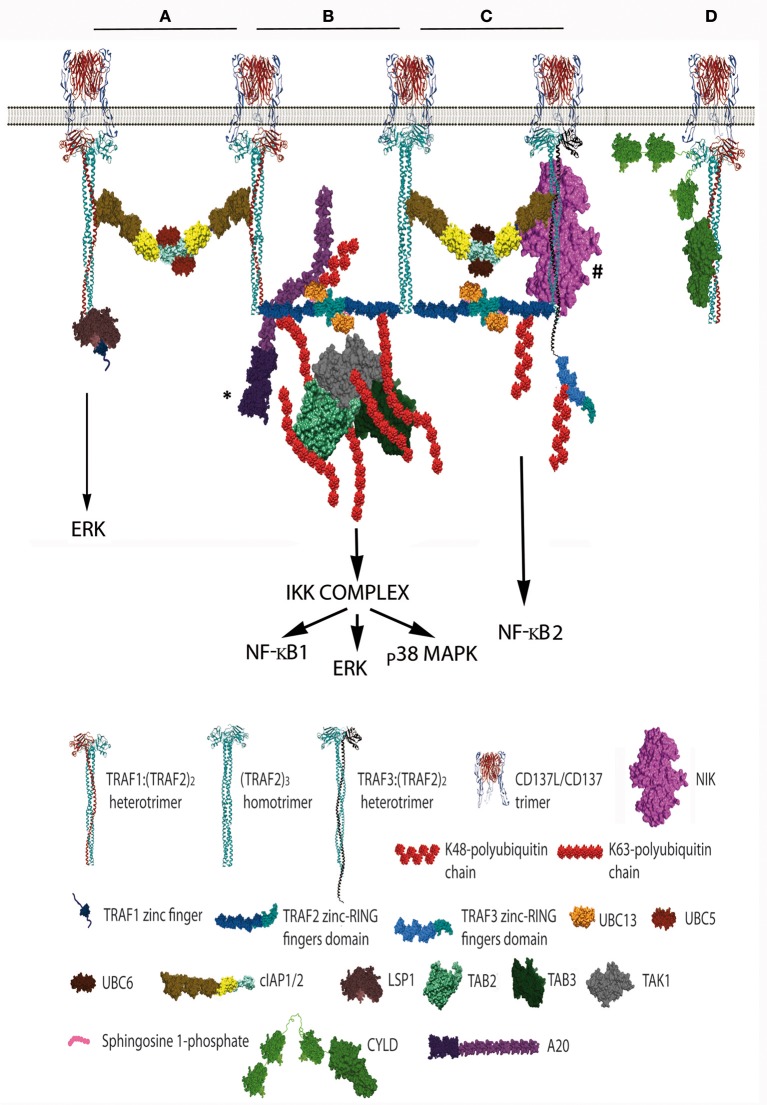
Schematic representation of the distinct CD137 signalosomes that would be formed upon CD137 activation. This figure illustrates the distinct signalosomes that could be formed in response to CD137 activation depending on the TRAF trimer configurations that get associated to the activated CD137. **(A)** cIAP1/2 bridging between 2 TRAF1(TRAF2)_2_ trimers. What other molecules, besides E2 proteins, would be specifically recruited to this configuration is yet unknown. The binding of Lymphocyte specific protein-1 to the N-terminal region of TRAF1 is shown. **(B)** The formation of a dimer between the RING finger domains of 2 TRAF2 molecules from adjacent trimers will trigger K63 ubiquitination of TRAF2 and the subsequent recruitment and activation of the TAK1/TAB1/TAB2/TAB3 complex (TAB1 is not shown). K63-TAK1-mediated IKKβ phosphorylation will activate the IKK complex activation initiating a signaling cascade that will result in NF-κB1 and ERK activation. A20 might inhibit this signaling cascade by K48-ubiquitinating Ubc13 thus inhibiting TRAF2 E3 ubiquitin ligase activity. ^*^ A20 can form dimers, but a sole A20 molecule is represented for clarity. **(C)** Hypothetical organization of a signalosome that includes a TRAF3:(TRAF2)_2_ trimer. The cIAP1/2 molecules associated either to a TRAF2 homotrimer and the hypothetical TRAF3:(TRAF2)_2_ trimer will form a dimer by the interaction of their RING fingers domains causing the activation of the E3 ubiquitin ligase activity. Thus, the cIAP1/2 dimer will K48-ubiquitinate TRAF3 and TRAF2 molecules targeting them for proteasome degradation and effectively releasing NIK from its interaction with TRAF3, resulting in the activation of NF-κB2 as has been observed following CD137 stimulation. # The TRAF region binding to NIK is still controversial, since reports indicating that is mediated by either the TRAF domain (56, 57) or the RING-zinc finger region (58, 59) are available. **(D)** CYLD interacts with the same crevice in the TRAF domain that binds to CD137 cytosolic tail. CYLD might works as a gate keeper preventing ligand-independent TRAF activation but it might also participate in the termination of CD137 signaling by outcompeting CD137 from binding to TRAF2 as shown in the figure. Further explanation in the text. Protein structure coordinates were obtained from the PDB database and molecular graphics were performed with UCSF Chimera (15). When this information was absent for a protein of interest, we modeled the proteins according to their domains using available structures of similar domains to provide an approximate representation of the protein structure and size.

Since the five zinc fingers and the RING finger domains of each TRAF2 molecule in the trimers will likely emanate from the intertwining coils in opposite directions (28, 64) and the active E3 ubiquitin ligase requires the formation of RING-finger dimers (65), TRAF2-RING finger dimers will likely be formed by the RING finger domains of two TRAF2 molecules from adjacent trimers, similar to what has been described for TRAF6 (66) (Figure 1). This inter-trimer bonding would help the clustering and stabilization of the two-dimensional hexagonal lattice (28, 64). In this case, since TRAF1 lacks of a RING finger domain, the presence of one TRAF1 molecule in a trimer would impede the formation of one intertrimer bonding but would not have any effect on the ability of the 2 TRAF2 molecules in the trimer to establish these TRAF2-RING dimers with neighboring TRAF2-containing trimers (Figure 1). The TRAF2-RINGs now in their active dimeric form will bind S1P and Ubc proteins (Ubc13 or UbcH5A) (Figure 2), getting ready to catalyze the K63-ubiquitination of TRAF2 itself and other target proteins (50). In addition TRAF2 trimers and TRAF1:(TRAF2)_2_ heterotrimers, but not TRAF1 trimers, will recruit a single cIAP1/2 (33). Indeed, cIAP1/2 will interact through its BIR1 domain (67) with the TRAF trimers by asymmetrically engaging two cIAP-interacting motifs in the coiled coil of two TRAF molecules in the trimer (33, 42). Of note is that cIAP1/2 interaction with the TRAF1:(TRAF2)_2_ heterotrimers is stronger than that with TRAF2 trimers and, therefore, cIAP1/2 would preferentially be bond to the TRAF1:(TRAF2)_2_ heterotrimers. Interestingly, TRAF1 homotrimers have a cIAP2 dissociation constant two orders of magnitude weaker than that of TRAF2 homotrimers, effectively precluding the interaction of cIAP2 with TRAF1 homotrimers (33). The interaction of cIAP1/2 BIR1 domain with TRAFs would release the cIAP-RING from its inhibitory interaction with the cIAP-CARD domain (68), allowing the formation of cIAP1/2-RING dimers and the binding of the E2 ubiquitin ligases. Since only one cIAP1/2 molecule associates to a TRAF trimer, the cIAP1/2-RING dimer would have to be formed by two cIAP1/2 molecules each one associated to adjacent TRAF trimers in the hexagonal lattice (Figures 1, 2), thus further bridging two neighbored activated TNFR complex. Altogether, these results indicate that albeit the basic signaling brick in CD137 (as well as of other TNFRs) would be a trimer, a trimer alone will not be able to signal as it seems absolutely necessary to establish inter-trimer bridging and multi-trimer clustering to build a functional signalosome (Figures 1, 2).

In this regard, it is worth mentioning that since TRAF1 lacks RING finger domain and TRAF1 homotrimers cannot recruit cIAP1/2 to the CD137 signalosome, CD137-associated TRAF1 homotrimers would fail on bridging adjacent trimers through the formation of RING-dimers, which might result in the disruption of the hexagonal CD137 network and the inhibition of the signaling. While this scenario might provide a rationale for the TRAF1-mediated inhibitory effects on some members of the TNFR family (43), many evidence support a positive role for TRAF1 in CD137 signaling (37, 47–49, 69). Therefore, other proteins interacting with TRAF1 might not only provide new functionality to the signalosome but also might contribute to the clustering of the activated CD137 receptors. In this regard, it has been shown that recruitment to CD137 of leukocyte-specific protein-1, a protein involved in CD137-mediated ERK activation, is mediated by its interaction with TRAF1 (70) (Figure 2A). Furthermore, Watts and coworkers (71) have shown that the TRAF-domain of TRAF1 directly interacts with three components of the linear ubiquitination (LUBAC) complex, SHARPIN, HOIP, and HOIL-1. In addition, Greenfeld and coworkers (41) have shown that TRAF1 is a key component of the Epstein-Barr virus Late Membrane Protein (LMP)-1 signaling complex, a protein that mimics TNFRs and uses TRAF proteins as scaffold (72). In this model, LMP1 promoted the association between TRAF1 and LUBAC and stimulated the linear M1-linked poly-ubiquitination of TRAF1, thus allowing TRAF1-mediated recruitment of the M1-ubiquitin binding proteins IKKγ and deubiquitinase (DUB) A20 (41) (see below). TRAF2 was essential for both LUBAC interaction and M1-polyubiquitination of TRAF1 (41), strongly suggesting the participation of TRAF1:(TRAF2)_2_ heterotrimers in this activity. In addition, binding of cIAP1 (73) and CYLD (74) to HOIP has also been reported. Although there is no evidence to date implicating LUBAC and M1-ubiquitination in the regulation of CD137 signalosome, research on this issue is warranted.

Soon after ligand activation, the growing CD137 signalosome gets decorated with K63-ubiquitinated proteins, mostly composed by K63-TRAF2 (36). Polyubiquitin chains linking the carboxyl terminus of ubiquitin molecules to the K63 of the next ubiquitin are well known as docking sites for downstream signaling components, and are required for building an effective signalosome (75–77). This is opposite to the polyubiquitination at K48, which in most cases targets proteins for proteosome-mediated degradation (77). TRAF2, associated to Ubc13 or UbcH5A, seems to be the main responsible of its own K63-ubiquitination (50) although cIAP1/2 associated to UbcH5A or Ubc13 also can catalyze K63-ubiquitination (78, 79). The next component of the CD137 signalosome getting recruited by K63-polyubiquitinated TRAF2 is a kinase complex composed by the transforming growth factor beta-activated kinase (TAK)-1 and TAK binding proteins (TAB)-1, 2 and 3 and (Figure 2B). Once recruited, TAK1 will get K63-ubiquitinated by TRAF2 (80). Taking lessons from the mechanism recently described for TRAF6-mediated TAK1 activation (81), efficient TRAF6-mediated TAK1 activation requires the synthesis of long K63-polyubiquitin chains by TRAF6. These long K63-polyubiquitin chains would have to be recognized by TAB2 and 3 (82) irrespective of whether they remain conjugated to TRAF6 or been unanchored (81). Interestingly, A20 which is a component of the CD137 signalosome (see below), effectively removes long K63-linked polyubiquitin chains from TRAF6 without disassembling the chains themselves (83). Once activated, TAK1 will phosphorylate the inhibitor of nuclear factor κ-B kinase (IKK)-β leading to the activation of canonical NF-κB (75) and ERK1/2 (48). TAK1 will also induce the activation of mitogen activated kinases kinases (MKK) MAP kinases, leading to p38MAPK activation (84).

While ubiquitination is a chief mechanism controlling TRAF-mediated CD137 signaling, the regulation of TRAF activity by phosphorylation has also been described. In this regard, it has been shown that TRAF2 phosphorylation at T117 by PKC promotes both K63-ubiquitination of TRAF2 and the recruitment of the IKK complex to activated TNFRs (85). Moreover, TRAF1 phosphorylation at S139 by TANK-binding kinase inhibits NF-κB activation in response to CD137 engagement (86).

Besides the induction of the canonical NF-κB pathway by CD137, the activation of the alternative NF-κB pathway by this TNFR has also been reported (38). The molecular mechanism controlling NF-κB2 is different to that controlling the canonical NF-κB1 pathway. In non-activated cells, NF-κB2 activation is prevented by continuous NF-κB-inducing kinase (NIK) degradation by a complex formed by TRAF2/cIAP and TRAF3/NIK. In non-activated cells, this complex works promoting cIAP1/2 mediated K48-ubiquitination of NIK and its subsequent proteasome-mediated degradation. However, upon TNFR activation, binding of this complex to the receptor results in cIAP-dependent degradation of TRAF3 (and often also of TRAF2), releasing NIK and allowing p100 processing to the active p52 NF-κB subunit [reviewed in (87)]. Thus, the induction of NF-κB2 by CD137 (38) implies that TRAF3 and NIK would have to be recruited to the CD137 signalosome (Figure 2C). In support of this event, it has been shown that TRAF3 is degraded upon CD137 engagement (38). However, it is still unclear whether TRAF2 and TRAF3 would be recruited as homotrimers to adjacent CD137 trimers or as TRAF2/TRAF3 heterotrimers to one ligand-activated CD137 trimer. In this regard, there is evidence suggesting the existence of TRAF2/TRAF3 heterotrimers (88). In addition, it is noteworthy that TRAF1 has been shown to directly interact with NIK, suggesting that TRAF1:(TRAF2)_2_-cIAP1/2 complexes can also be a component of the E3 ubiquitin ligase complex for NIK (33, 89). However, there are conflictive results on whether TRAF1 is an activator or an inhibitor of the NF-κB2 pathway. It has been proposed that the binding of TRAF1 to NIK causes the disruption of TRAF2:cIAP1/2 binding, resulting in NIK stabilization and NF-κB2 activation (89). However, studies on the role of TRAF1 in CD137-mediated NF-κB activation show that in the absence of TRAF1, NF-κB1 induction is restricted while NF-κB2 induction proceeds more efficiently (38). These results might indicate that the tighter association of cIAP1/2 to the TRAF1:(TRAF2)_2_ heterotrimers compared to that of the TRAF2 homotrimers might restrict the ability of cIAP1/2 to shift their targets from NIK to TRAF3. Besides, it is also conceivable that an overabundance of TRAF1 might interact with all available TRAF2 molecules, thus precluding the formation of the TRAF2:TRAF3 heterotrimers. Interestingly, and as described above, TRAF1 protects TRAF2 from degradation (45, 46). Whether this protection could be caused by the inability of cIAP1/2 in the TRAF1:(TRAF2)_2_ heterotrimers to K48-ubiquitinate TRAF2 while it could do it as part of the TRAF2 homotrimers deserves further investigation.

Adding just another level of complexity to an already crowded CD137 signalosome, we have recently observed the functional association of K63-DUBs A20 and CYLD to the CD137 signalosome. This interaction results in the downregulation of CD137-elicited K63-ubiquitination and signaling toward NF-κB activation in both primary T cells and transfected cell lines (90). A20 was first described as an ubiquitin-editing enzyme (91). It is composed of an N-terminal ovarian tumor (OTU) domain, which would catalyze the removal of K63-ubiquitin chains from target proteins, and a C-terminal zinc-finger domain region, which endows this protein with E3 ubiquitin ligase activity able to transfer K48-polyubiquitin chains to those target proteins, thereby promoting its proteasome-mediated degradation (92). However, recent evidence shows that A20 can efficiently remove K48-linkages but is almost inactive toward K63-linkages, raising questions on what is the actual mechanism by which A20 inhibits the NF-κB pathway [reviewed in (93)]. Interestingly, A20 is also able to inhibit K63-ubiquitination by promoting K48-ubiquitination and degradation of E2 ligases, such as Ubc13 and UbcH5C, thus effectively inhibiting the E3 ligase activity of TRAF2, cIAP1/2, and TRAF6 (94) (Figure 2B). In addition A20 can also inhibit NF-κB by interacting with and sequestering Nemo (IKKγ), thus impeding IKKβ activation without requiring the DUB and E3-ubiquitin ligase activities of A20 (95–97).

CYLD is another member of the DUB family. It contains three cytoskeletal-associated protein (CAP)-glycine conserved repeats at the N-terminus and a DUB domain at its C-terminus. CYLD has a TRAF-interacting motif and has been shown to interact with TRAF2 and to catalyze the removal of TRAF2-linked K63-ubiquitin chains, precluding IKK from being activated (98). Interestingly, phosphorylation of CYLD by IKK inhibits its DUB activity (99). This result opens the possibility that CYLD might works as a gate keeper preventing ligand-independent activation, and that once receptor signaling unlocks, CYLD would be kept inactive by the active IKK complex. Since CYLD has also been found associated to the CD137-signalosome (90), this suggests that CYLD might also participate in the termination of CD137 signaling by outcompeting CD137 from binding to TRAF2 (Figure 2D). Altogether, these results underscore the relevance of the ubiquitination and deubiquitination processes in the regulation of CD137 signaling, evidencing that the balance between K63- and K48-ubiquitination of key target proteins will determine the outcome of the response. A summary of the role of TRAF1-3 in controlling CD137 signal transduction and function is provided in Table 1.

**Table 1:** Role of TRAF1,2 and 3 in CD137-mediated signaling.

	**^TRAF1^**	**^TRAF2^**	**^TRAF3^**
TRAF heterotrimers	TRAF2 (33)	TRAF1 (33) TRAF3 (suspected)	TRAF2 (suspected)
Ubiquitin ligase activity	no	K63 (49–52) K48 (53, 54)^*^	unknown
Substrates	no	TRAF2 (50) TAK1 (80)	unknown
Binding partners	cIAP1/2 (33) LSP-1 (70) NIK (33, 89) LUBAC (undetermined)	cIAP1/2 (33) ubcl3 (50) CYLD (98)	NIK (87)
Functional data	CD137-mediated survival of memory T cells (49). CD137-mediated antivirus responses (37, 49) Required for activity of CD137-based chimeric antigen receptors (CARs) (39)	CD137-mediated costimulatory signaling in T cells (32) CD137-mediated tumor rejection in xenograft mice (36). Required for activity of CD137-based chimeric antigen receptors (CARs) (39).	Required for activity of CD137-based chimeric antigen receptors (CARs) (39)

The table summarizes the known function and binding partners of these TRAF proteins in the regulation of CD137 activity,as well as the available in vivo or ex vivo T cells functional data.

* *not demonstrated in CD137 signaling*.

Finally, we have observed that upon ligation with anti-CD137 antibodies, CD137 signalosome becomes internalized and is transferred to an endosomal compartment in a K63-polyubiquitin-dependent manner (36). Nam and coworkers (34) showed that CD137 engagement caused its redistribution into lipid rafts, in a process that seems to be dependent on TRAF2 binding to Caveolin (100) and Filamin A (101), which are intrinsic components of the lipid rafts. Thus, the CD137 signalosome-containing endocyted vesicles might be caveolae that later fuse with early endosomes (102), but this awaits confirmation. Interestingly, So and Croft (103) have proposed that TRAF2-dependent recruitment of activated CD137 into lipid rafts might be behind the observed activation of PI3K-AKT signaling pathway by CD137. Lipid rafts are membrane microdomains that facilitate AKT recruitment and activation upon phosphatidylinositol-3,4,5-triphosphate accumulation in the plasma membrane (104). The mechanism involved in CD137-mediated PI3K/AKT activation is still unknown, although its relevance in promoting CD137-mediated T cell proliferation and apoptosis protection seems well sustained (105–107). As there is no evidence of a direct association of PI3K and/or AKT to the CD137 signalosome, PI3K-AKT ought to be activated by other signaling complexes, such as TCR/CD28, working together with CD137 (105, 107). Since activated TCR/CD28 reside in the lipid rafts, these lipid structures might work as multi-signaling hot-spots (103). Indeed, it would be plausible that the ligand-activated CD137 hexagonal lattice keeps trapped inside (in the center of the hexagons) TCR and CD28 complexes that would move together with the activated CD137 trimers to lipid-rafts, thus facilitating the response to antigen. However, since CD137-mediated AKT activation is delayed compared to that of ERK and NF-κB, taking hours instead of minutes (107), efficient CD137-mediated triggering of PI3K/AKT activity may require additional players (whose expression might even be induced by CD137 engagement) and/or further signaling-complexes compartmentalization to proceed.

Interestingly, we have observed that endocyted CD137 signalosome-containing vesicles remain decorated with K63-polyubiquitin chains, strongly suggesting that CD137 signaling is still active during this process (36). However, it is expected that the endosomes will later fuse with lysosomes to recycle its content. Interestingly, it has been shown that A20 can target TRAF2 to the lysosome for its degradation, which is dependent on the membrane tethering activity of A20, but not of its ubiquitin-modifying function (108). These CD137-mediated endocytosis experiments (36) were performed with agonist anti-CD137 mAbs and, therefore, it is yet to be determined whether CD137 engagement with CD137L would also cause the internalization of the complex, but it is likely that this will actually occurs for various reasons. First, CD137 internalization has been already observed in dendritic cells upon binding to CD137L fusion proteins used to target antigens for vaccination (109). Second, an accumulation of CD137 on the surface of *CD137L*-deficient T cells has been observed, probably as a result of the impossibility of CD137 to be internalized in the absence of CD137L (110). Third, because many key molecules in CD137 signaling, such as CD137, TRAF1, TRAF2 and cIAP1/2 are readily transcriptionally activated by NF-κB and AP1 transcription factors upon CD137 activation (111–114), restocking these molecules and ensuring CTL responsiveness to new CD137 costimulatory rounds. Finally, because it has been recently described that tonic chimeric antigen receptor (CAR)-derived CD137 signaling causes T cell toxicity by the continuous TRAF2-mediated NF-κB activation and increased Fas-dependent cell death (115). This result emphasizes the deleterious effects that unrestricted CD137-signaling would have in the cells and underscores the key role of the multiple mechanisms controlling CD137 signaling described above, including CD137 internalization.

## Understanding CD137 signaling to improve CD137-mediated immunotherapy

CD137 has become one of the most relevant molecular targets in cancer immunotherapy for its ability to drive CTL and NK cells anti-tumor responses. Humanized anti-CD137 mAbs have entered the clinic (21). One of those (urelumab) showed promising anti-tumor effects as a monotherapy treatment in a phase I trial. Unfortunately, a follow-up Phase II trial revealed severe liver toxicity in a significant number of patients (10%) that resulted in two fatalities (116). Consequently, trials with urelumab as a monotherapy were terminated (117). A comprehensive safety analysis of patients treated with urelumab confirmed a strong association between hepatitis and the urelumab dose and resulted in dose reductions in subsequent clinical trials (118). In this regard, ongoing clinical trials with urelumab and other anti-human CD137 mAbs used in combinatory therapies are underway. Alternative approaches are needed to circumvent the off-target toxicity associated to these treatments while preserving their efficacy, for instance, by targeting these agonist antibodies or the natural ligand to surface molecules expressed on cells present in the tumor microenvironment (21). Another important strategy to improve anti-CD137 mAb anti-tumor activity, while limiting its side effects, would be boosting CD137-mediated signal transduction. Many aspects of CD137 signaling might be of interest for drug development, including interfering with negative regulators of CD137 signaling, promoting optimal complex/scaffold formation, and keeping signaling-CD137 endosomes from lysosome degradation, among others. These approaches have been neglected so far due to our limited understanding of the different mechanisms controlling CD137 signal transduction, a limitation that could also be extended to other members of the TNFR family.

These limitations would also apply to the usage of CD137 signaling for enhancing chimeric antigen receptor (CAR) T cell effectiveness. In this regard, transducing T cells with a CAR-construct containing the CD137 cytosolic tail together with the CAR-CD3ζ proved to be effective in increasing CTL cell survival, targeting of CTLs to the tumor and boosting anti-tumor activities (119). Remarkably, it has been recently demonstrated the clinical effectiveness of this therapy in the treatment of relapsed or refractory B-cell acute lymphoblastic leukemia (120, 121).

In the case of CD137 containing CARs, recent evidence shows that the activity of CD19-targeted CAR T cells with a CD137 endodomain is dependent on TRAF1, 2 and 3 and also on NF-κB activation (39). However, little is known on whether the molecular mechanisms controlling the extent of the response are similar to those of native CD137. In this regard, and as stated above, tonic chimeric antigen receptor (CAR)-derived CD137 signaling has been shown to cause T cell toxicity by the continuous TRAF2-mediated NF-κB activation and increased Fas-dependent cell death (115), thus highlighting the need of a better understanding of the molecular mechanisms controlling CD137 signaling. Moreover, the development of CAR T cells with CD137 intracellular tail acting in tandem with the cytoplasmic domain of CD3 ζ may promote a signaling crosstalk between these 2 pathways. Interestingly, and as discussed above, this crosstalking between CD137 and the TCR might be happening at certain extent in normal CD137-signaling (103). In any event, the CD137 component in the CAR T therapy is key to ensure the functional persistence and survival of the transduced T cells (119, 120) a feature ultimately needed for clinical efficacy, but also keeping in mind that unrestrained CD137 activity might also be deleterious for the cell (115). Translational research in the signal transduction pathways controling CD137-mediated responses should focus in the identification of druggable targets that would allow toning up or toning down CD137 activity as needed. In addition, developing tools for early and reliable detection of CD137-signaling events and/or their outcome would be paramount to define pharmacodynamic biomarkers and useful parameters to optimize new generations of CD137 agonists.

## Author contributions

JZ, GP-C, PC-B, IM-F, AA, IO, and IM critically revised the manuscript for important intellectual content. JZ, GP-C, PC-B, and IM designed the figures. JZ and IM wrote the paper.

### Conflict of interest statement

IM has served as a consultant for Bristol-Myers Squib, Roche-Genentech, Bayer, Alligator, Tusk, Bioncotech, Medimmune, Genmab, F-Star. IM receives commercial grants from Bristol Myers Squibb, Roche-Genentech and Alligator. IM-F is a full time employee for MSD. The remaining authors declare that the research was conducted in the absence of any commercial or financial relationships that could be construed as a potential conflict of interest.
